# Analysis of Circulating Tumor Cells in Patients with Non-Metastatic High-Risk Prostate Cancer before and after Radiotherapy Using Three Different Enumeration Assays

**DOI:** 10.3390/cancers11060802

**Published:** 2019-06-10

**Authors:** Joanna Budna-Tukan, Monika Świerczewska, Martine Mazel, Wojciech A. Cieślikowski, Agnieszka Ida, Agnieszka Jankowiak, Andrzej Antczak, Michał Nowicki, Klaus Pantel, David Azria, Maciej Zabel, Catherine Alix-Panabières

**Affiliations:** 1 Department of Histology and Embryology, Poznan University of Medical Sciences, 60-781 Poznan, Poland; mswierczewska@ump.edu.pl (M.Ś.); agajankowiak@hotmail.com (A.J.); mnowicki@ump.edu.pl (M.N.); 2 Laboratory of Rare Human Circulating Cells (LCCRH), University Medical Centre of Montpellier, 34093 Montpellier, France; martine-mazel@chu-montpellier.fr; 3 Department of Urology, Poznan University of Medical Sciences, 61-285 Poznan, Poland; w.cieslikowski@gmail.com (W.A.C.); agnieszka.ida@gmail.com (A.I.); aa26@poczta.onet.pl (A.A.); 4 Department of Tumor Biology, University Medical Centre Hamburg-Eppendorf, 20246 Hamburg, Germany; pantel@uke.de; 5 Radiation Oncology Department, Montpellier Cancer Institute, 34298 Montpellier, France; david.azria@icm.unicancer.fr; 6 Division of Histology and Embryology, Department of Human Morphology and Embryology, Wroclaw Medical University, 50-368 Wroclaw, Poland; mazab@ump.edu.pl; 7 Division of Anatomy and Histology, University of Zielona Góra, 65-046 Zielona Góra, Poland

**Keywords:** liquid biopsy, CTC, prostate cancer, early diagnostic, PSA, radiotherapy

## Abstract

The characterization of circulating tumor cells (CTCs) can lead to a promising strategy for monitoring residual or relapsing prostate cancer (PCa) after local therapy. The aim of this study was to compare three innovative technologies for CTC enumeration in 131 high-risk patients with PCa, before and after radiotherapy, combined with androgen deprivation. The CTC number was tested using the FDA-cleared CellSearch^®^ system, the dual fluoro-EPISPOT assay that only detects functional CTCs, and the in vivo CellCollector^®^ technology. The highest percentage of CTC-positive patients was detected with the CellCollector^®^ (48%) and dual fluoro-EPISPOT (42%) assays, while the CellSearch^®^ system presented the lowest rate (14%). Although the concordance among methods was only 23%, the cumulative positivity rate was 79%. A matched-pair analysis of the samples before, and after, treatment suggested a trend toward a decrease in CTC count after treatment with all methods. CTC tended to be positivity correlated with age for the fluoro-EPISPOT assay and with PSA level from the data of three assays. Combining different CTC assays improved CTC detection rates in patients with non-metastatic high-risk PCa before and after treatment. Our findings do not support the hypothesis that radiotherapy leads to cancer cell release in the circulation.

## 1. Introduction

The management of patients with prostate cancer (PCa) is challenging because more than 40% of surgically treated patients will experience a biochemical disease recurrence [1]. Moreover, according to D’Amico criteria, 15% of PCa patients are already high risk at diagnosis, and are defined as clinical T stage ≥cT2c, a Gleason score ≥8, or a prostate specific antigen (PSA) >20 ng/mL [2]. Local therapy, such as radical prostatectomy and/or radiation, is complex because PCa heterogeneous phenotypes lead to different clinical outcomes, ranging from indolent to lethal metastatic diseases [3]. Thus, PCa diagnosis is a crucial issue as early disease detection significantly increases recovery rate and overall survival. The most commonly used diagnostic tools, such as serum PSA level, transrectal ultrasonography (TRUS), Gleason histologic grading of biopsy specimens, and the clinical tumor, node, metastasis (TNM) staging, do not allow for the precise determination of each patient’s current tumor status [1]. Indeed, serum PSA level does not unequivocally differentiate between aggressive and indolent cancer, and can be affected by several factors, such as age or urinary tract infections [4]. Moreover, the reliability of PSA testing for PCa screening is insufficient because patients with PSA lower than 4.0 ng/mL can have PCa [5]. Biopsy analysis can give false-negative results, caused by small tumor size or uneven distribution of cancer cells within the tumor [6]. Hence, novel, specific, and improved prognostic markers are needed to determine clinically significant tumors causing progressive disease, and this information can then be implemented into personalized treatment of cancer patients at early disease stages [7,8].

The characterization of circulating tumor cells (CTCs) is a new promising method to understand the biology of residual or relapsing PCa after local therapy. As CTCs are shed into the bloodstream during primary tumor growth and progression, they can be used to predict disease progression, select therapeutic targets, and monitor therapy outcome [9]. It has been shown that CTC count is proportional to survival in metastatic breast, colorectal, and prostate cancer [10]. In patients with non-metastatic cancers (TNM-stage M0), including PCa, the clinical relevance of CTC counts is still under investigation. The strongest data sets exist for breast cancer, demonstrating independent prognostic relevance in patients receiving (neo-)adjuvant chemotherapy [11,12,13,14].

The major challenge in CTC research is the isolation of these very rare cells (typically one single CTC in 10^6^–10^7^ leukocytes from peripheral blood of patients with cancer) [15]. A number of new innovative technologies, that improve CTC detection, have recently been described. The most widely used approach is based on immunoaffinity, where cells are captured by targeting tumor-associated antigens (positive enrichment). They include immunomagnetic assays, like CellSearch^®^ [16], Adna Test [17], or MACS [18], microfluidic devices like CTC-Chip [19], Herringbone Chip [20], and spiral ClearCell^®^ FX chip [21] or finally combination of mentioned above, like Ephesia [22], LiquidBiopsy [23] and IsoFlux [24]. On the contrary, some technologies use antibodies against leukocyte-associated antigens (negative enrichment), i.e., EasyStep Human CD45 Depletion Kit [25] or QMS [26]. Among them, the EpCAM-dependent CellSearch^®^ system, which is the only method currently approved by the Food and Drug Administration (FDA) for CTC selection and enumeration, is regarded as the “gold standard” and used for monitoring metastatic breast, colon, and prostate cancer [10]. Studies in patients with PCa showed a correlation between CTC number and clinical outcome [27].

However, CTC detection still faces many problems. Cancer heterogeneity seems to be the major obstacle because even cancer cells derived from the same tumor can present various immunophenotypes. Epithelial to mesenchymal transition (EMT) is considered a crucial reason for the change. During this process the expression of epithelial markers, such as EpCAM and/or cytokeratins can be significantly downregulated and may limit CTC detection by EpCAM-dependent technologies, like CellSearch^®^ [28]. This is the rationale to combine EpCAM-dependent with EpCAM-independent technologies. Another limitation of the “gold standard” system is the fact that the targeted epithelial phenotype (EpCAM- and cytokeratin-positive) does not mirror the tumor-specific phenotype. Thus the cytokeratin-negative, non-epithelial sub-population of CTCs, is lost during the analysis, which is restricted to the initial biomarker selection [29,30]. Moreover, CellSearch^®^ presents quite a high rate of apoptotic cells captured [31], and which cannot determine whether the detected CTCs are viable and able to form metastases. The fluoro-EPISPOT assay (Epithelial ImmunoSPOT, CHU Montpellier patent 2002), based on the detection of CTC-secreted proteins, is a promising alternative for the detection of functional CTCs because only viable cells can secrete, shed or release proteins [32]. Furthermore, in vivo CTC isolation techniques, which significantly increase the volume of blood tested, have gained significant attention. Among them, the in vivo CellCollector^®^ has been used in clinical studies on PCa and other solid tumors [33,34].

In this study, we compared these three techniques for CTC detection in patients with high-risk PCa treated by radiotherapy (RT). Among them only the automated CellSearch^®^ system has already been studied by many different groups all over the world. For prostate cancer, worldwide there are only five reports of the EPISPOT assay being used [32,34,35,36,37] and three reports using the CellCollector^®^ [34,37,38], with two in each group being a component of our multicenter ERA-NET TRANSCAN study. To the best of our knowledge, there are no reports using these two assays on patients with non-metastatic prostate cancer receiving radiotherapy. We can demonstrate that the combined use of these three assays increased the detection rate of CTCs in patients with non-metastatic PCa, that are known to have very low CTC concentrations [15]. To evaluate the potential clinical relevance of CTC detection, we analyzed the correlation between the CTC counts obtained with each method and conventional risk factors.

## 2. Results

### 2.1. Patient Characteristics

Between 2013 and 2016, 131 patients with non-metastatic high-risk PCa were recruited at diagnosis before any therapy (baseline), and 68/131 could be seen again after 256 (±126) days after the initiation of therapy (summary of the baseline clinical characteristics in Table 1). At baseline, their median age was 68.5 years, the median PSA concentration was 27.85 ng/mL, and the median Gleason score on biopsy was 7. During treatment, metastases were detected in 19 patients who were excluded from the statistical analysis.

The number of samples tested with each method at baseline and/or after treatment are listed in Table 2.

### 2.2. Comparison of CTC Detection Rate Obtained with Three Assays

**Figure 1:**
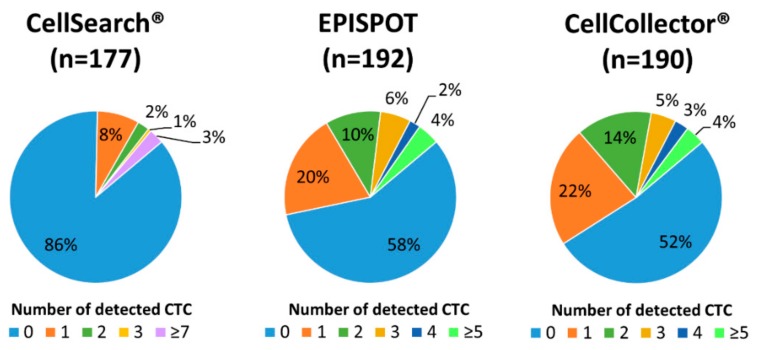
Distribution of detected circulating tumor cells (CTC) using the CellSearch^®^ system, dual fluoro-EPISPOT assay, and CellCollector^®^. The results of CTC quantification in all samples (i.e., samples collected one day before and at least three months after radiotherapy) tested with the indicated assay.

First, the total number of CTC-positive samples (baseline and after the treatment) obtained using each method was compared (Figure 1).

**Figure 2:**
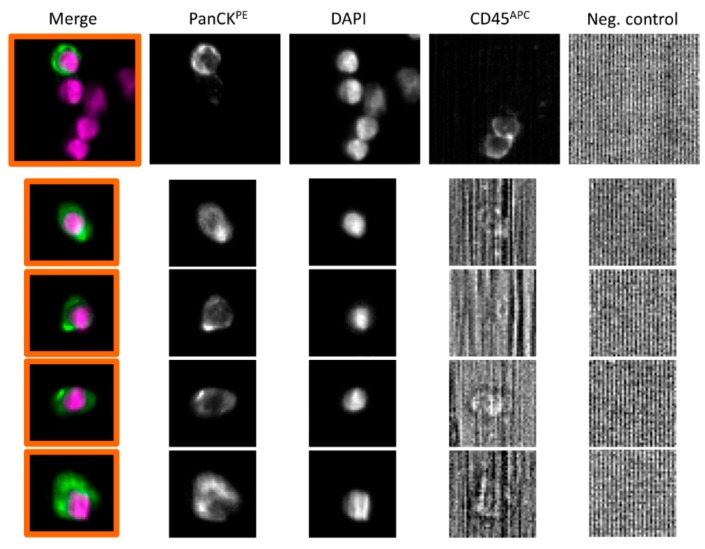
Representative images of CTCs from patients with high-risk PCa, that was detected using the CellSearch^®^ system. Cells were identified as tumor cells according to the following criteria: EpCAM-positive, panCK-positive, DAPI-positive, CD45-negative and negative for the last channel. CK: Cytokeratin; PanCK: anti-CK8, -18, -19 antibodies; PE: Phycoerythrin; APC: Allophycocyanin.

With the CellSearch^®^ system, 24/177 (14%) patient samples had ≥1 CTCs (range 1–54, median 1) (Figure 2 for representative images of PanCK-positive and CD45-negative CTCs). 

**Figure 3:**
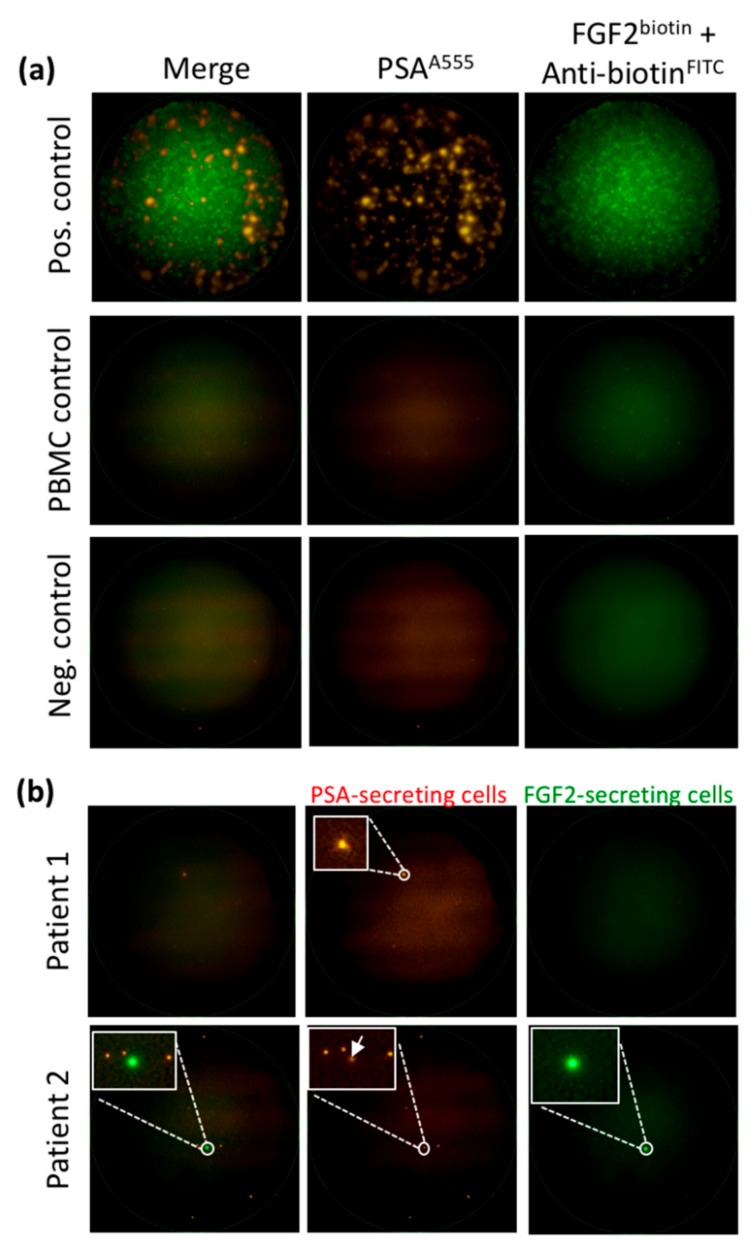
Detection of viable CTCs using the dual fluorescent EPISPOT^PSA/FGF2^ assay. (**a**) Positive and negative controls. LNCaP (shown in figure) and NBTII cells that secrete PSA and FGF2, respectively, were used as positive controls (2000 cells/well), whereas wells with peripheral blood mononuclear cells (PBMC) and without cells were used as negative controls. Each immuno-spot corresponds to the protein “fingerprint” of one viable cell. (**b**) Patient samples. PSA-secreting cells are considered as CTCs in blood samples from patients with PCa (Patient 1). A subset of CTCs can secrete FGF-2 in addition to PSA (Patient 2). Representative images of PSA-positive, FGF2-positive and double PSA/FGF2 immuno-spots (merge) corresponding to viable CTCs. Immuno-spots were detected and observed using the C.T.L. Elispot Reader, 50× magnification.

Using the dual fluoro-EPISPOT assay, which detects only viable PSA/FGF2-secreting CTCs, 81/192 (42%) patient samples had ≥1 CTCs (range 1-25, median 2) (Figure 1). Very few PSA-secreting CTCs also secreted FGF2 (9 out of 81) (Figure 3). Concerning the control groups for the EPISPOT assay in prostate cancer, we already reported data on the detection of PSA-secreting cells (defined as CTCs in prostate cancer) in patients with benign diseases (e.g., acute prostatitis, benign hyperplasia of prostate) as well as in healthy donors [39,40]; we have never observed any positive events in these two control groups. We also reported that PBMC from healthy donors do not secreted PSA nor FGF2 (Figure 3).

**Figure 4:**
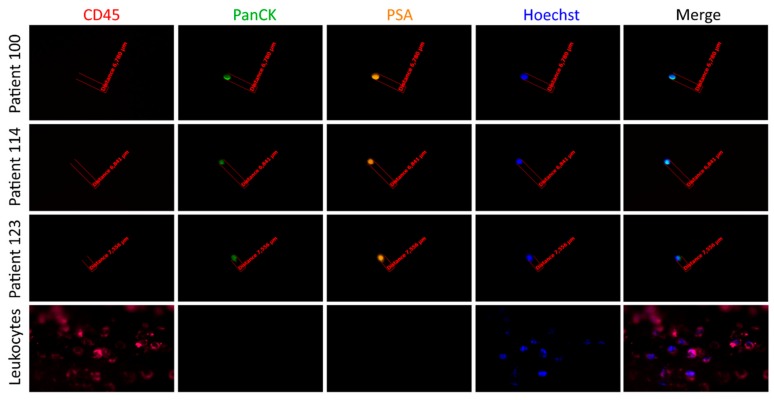
Detection of CTCs captured by the CellCollector^®^ in vivo system. Representative images of CTCs isolated in vivo and non-specifically bound leukocytes (CD45-positive). The cells were identified as tumor cells according to the following criteria: panCK-positive (green), CD45-negative (red) and optionally, PSA-positive (orange). The nucleus was stained with Hoechst 33258 (blue). The images were obtained using a fluorescent microscope (Carl Zeiss, Axio Imager 2, 20×) and analyzed with the Carl Zeiss, Axio Vision 4.8 software.

Finally, 91/190 (48%) patient samples had ≥1 CTCs (range 1–16, median 2) using CellCollector^®^ (Figure 1). Among the 197 PanCK-positive CTCs, 184 also expressed PSA (93%) (Figure 4).

**Figure 5:**
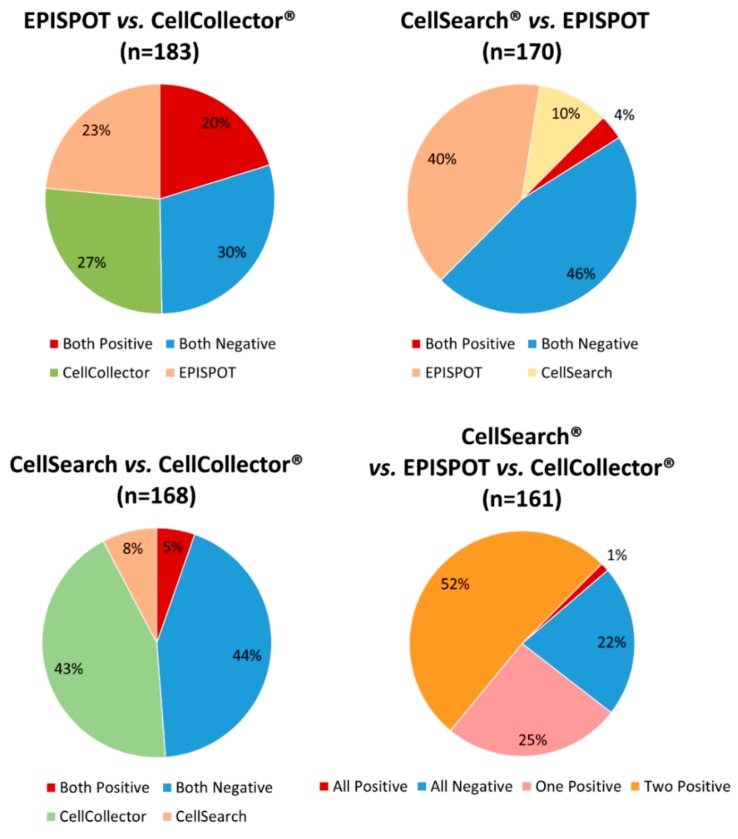
Concordance between two or three CTC detection assays. The chart shows the number of concordant positive (red) or negative (blue) results for the same sample obtained with two or three detection assays, as indicated.

By combining all the samples (baseline and after 256 (±126) days of treatment) assessed with the three methods (*n* = 161 samples), the number of positive samples (i.e., CTCs detected at least by one of the three assays) increased to 79% (127/161), with 8.7% (14/161) of blood samples harboring ≥ 5 CTCs. The concordance among the three assays (all positive or all negative) was 23%, while the concordance between two assays was approximately 50% for each comparison. The positive concordance was observed in samples with low number of CTCs, harboring up to 3 CTCs (Figure 5).

### 2.3. Analysis of Matched Blood Samples before and after Radiotherapy 

**Figure 6:**
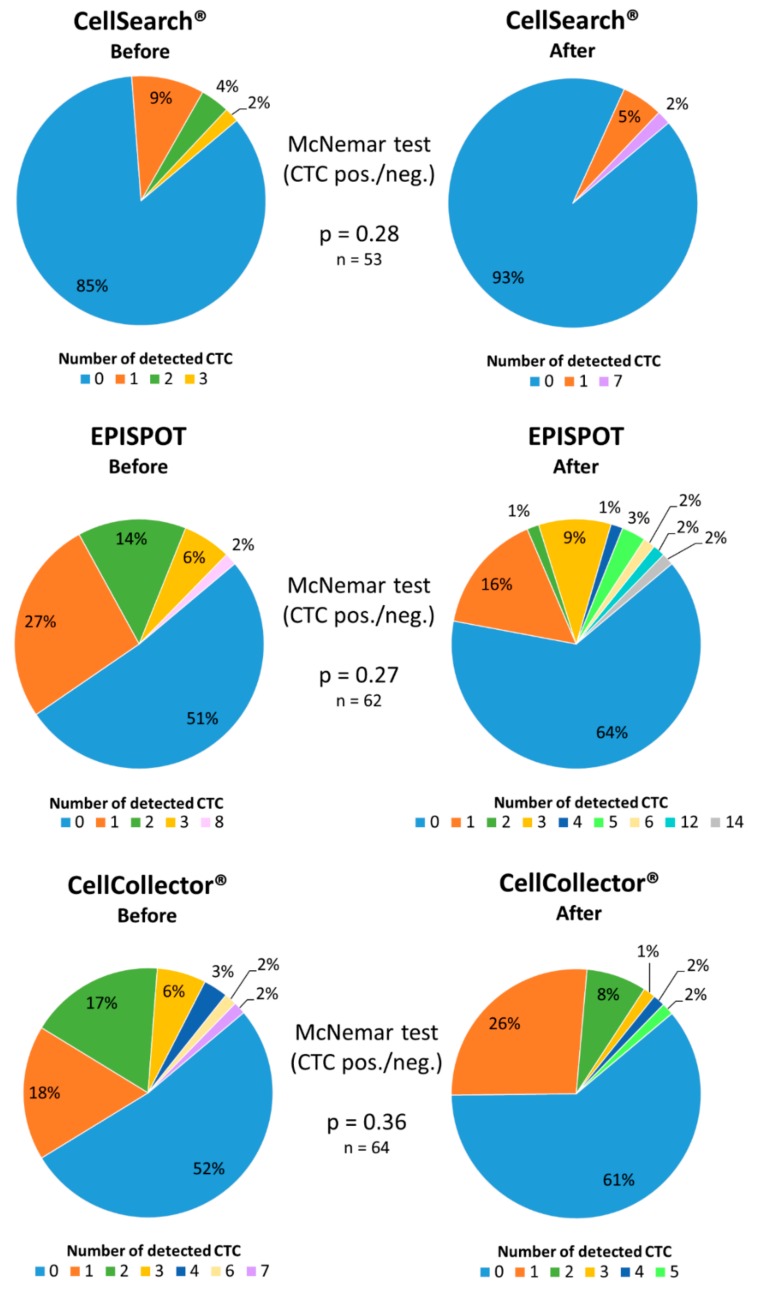
Matched-pair analysis of CTCs quantified with the CellSearch^®^, dual fluoro-EPISPOT^PSA/FGF2^ or CellCollector^®^ assay before and at least three months after radiotherapy start.

Matched-pair analysis of CTC counts before and after radiotherapy for the patients seen at both occasions (*n* = 68) did not find any significant difference for all three assays (CellSearch^®^ *p* = 0.28, dual fluoro-EPISPOT *p* = 0.27, CellCollector^®^ *p* = 0.36) (Figure 6). 

Nevertheless, CTC positivity rate tended to decrease in the samples collected after treatment. Specifically, using the CellSearch^®^ system, 15% of patients had a positive CTC count before, and 7% after treatment. Most of them (83%; 44/53) presented no change, whereas in 13% (7/53) CTC number was decreased and in 4% (2/53) increased at the second analysis. With the dual fluoro-EPISPOT assay, CTC positivity rate decreased from 49% before to 36% after the initiation of radiotherapy. Moreover, in 39% (24/62) of patients CTC number did not change between assays, whereas in 32% (20/62) decreased and in 29% (18/62) increased. With the CellCollector^®^ test, CTC positivity decreased from 48% before to 39% after radiotherapy; CTC number did not change between assays in 39% (25/64), decreased in 36% (23/64) and increased in 25% of patients.

### 2.4. Correlation Between CTC Count and Clinical Parameters

Analysis of the correlations between baseline CTC counts (each method separately) and clinical parameters (age, body mass index, PSA median level, Gleason score and clinical tumor stage) only found that patients with ≥1 CTCs (obtained with the dual fluoro-EPISPOT**^PSA/FGF2^**) tended to be younger than patients with negative CTC counts (*p* = 0.053) (Table 1). Finally, an analysis of the combined CTC counts (three methods together) showed a significant correlation between CTC counts and patients’ body mass index (*p* = 0.0036) (Table 1).

## 3. Discussion

In this study, we compared three different CTC identification/enumeration methods (CellSearch^®^, dual fluoro-EPISPOT**^PSA/FGF2^** assay and CellCollector^®^) in a cohort of patients with localized high-risk PCa, treated with radiotherapy, combined with androgen deprivation. A comparison of the results obtained with each assay showed that the CellSearch^®^ system presented the lowest CTC detection rate (only 14% of samples compared with 42% and 48%, with dual fluoro-EPISPOT**^PSA/FGF2^** and CellCollector^®^, respectively). Previous studies using CellSearch^®^ reported CTC positivity rates in patients with PCa that varied from 14% [41] to 37% [34], in non-metastatic prostate cancer patients. This observation, although unexpected, can be explained. CellSearch^®^ technology regarded as “gold standard” for CTC detection was cleared by FDA for metastatic cancers [10]. We applied CellSearch^®^ in our study on non-metastatic PCa patients, based on the available data describing its use in non-metastatic breast [42], colorectal [43], esophagus [44], and bladder [45] cancers. However, the concentration of CTCs in non-metastatic cancer patients is very low, and increasing the blood volume has helped to increase the rate of CTC detection in early stage breast cancer [11]. Interestingly, the EpCAM-based IsoFlux System, with only slightly higher blood volume suitable for testing (up to 10 mL), presented a very high CTC recovery rate in prostate and hepatocellular cancer patients. The IsoFlux System was designed to maximize the recovery of low EpCAM-expressing cells, specifically, those undergoing EMT or expressing stem cell markers through the application of another antibody cocktail. Additionally, the modification of magnetic bead coupling, flow rate, and microfluidic chamber dimension resulted in higher system sensitivity, compared to CellSearch^®^ [24,46]. Further studies, with increased number of patients, are required to fully evaluate IsoFlux System diagnostic usefulness. 

The positivity rates of the other two tests were comparable. Importantly, the EPISPOT assay uses only 10 mL of blood, which is comparable to the volume required by CellSearch^®^. Thus, the higher number of CTCs can be attributed to different principles of detection. Since the EPISPOT is an EpCAM-independent assay, the putative EMT process does not influence the detection outcome, which leads to a higher yield. However, the major novelty of the EPISPOT assay is the detection of proteins that are shed, actively released, or secreted by viable CTCs, which can contribute to tumor cell dissemination towards more distant sites [36]. Indeed, as not all circulating cells detached from a solid tumor are viable, the real risk of metastasis is hard to estimate on the basis of the count of all (dead and viable) CTCs. Our previous studies on breast cancer patients clearly indicated that patients with CK19-releasing viable CTCs showed significantly reduced overall survival rates compared to patients that do not harbor them. The stratification of breast cancer patients to low- and high-risk groups was improved when EPISPOT and CellSearch^®^ assays were combined [47]. The current EPISPOT technology is oriented against PSA protein product, being the biomarker of prostate-derived CTCs, and additionally the stem cell growth factor, FGF2, which was found to mediate the in vitro growth of micro-metastatic cells [48]. Corresponding analysis of prognosis prediction will be conducted with prospective clinical data of recruited PCa patients. The EPISPOT assay has been validated in patients with breast [49], colon [50], and prostate cancer [32]. Previous studies using this assay, in patients with localized PCa, reported CTC-positivity rates that ranged from 58.7% [34] to 71% [51].

The positivity rate obtained from the CellCollector^®^ in vivo capture system was slightly higher than the one from the EPISPOT assay, and the values were in the range of those reported by Kuske et al. [34] in patients with high-risk PCa, and by Gorges et al. [33], in patients with lung cancer. The main advantage of this tool is its ability to screen higher blood volumes by catching the CTCs directly from the blood. Clinical studies, with localized PCa patients, showed higher sensitivity and increased CTCs capturing rates, as compared to the CellSearch^®^ system [33]. Importantly, control tests on blood from healthy volunteers showed that no “CTC-like” events were found [33,52], proving the high specificity of the device. Additionally, molecular analysis on in vivo isolated CTCs is feasible, enabling in depth analysis of the mechanisms governing cancer progression and the optimization of treatment profiles [53]. However, the distinctive feature of in vivo CTC isolation brings some concerns as well. Compared to ex vivo CellSearch^®^ and EPISPOT technologies, the Cell-Collector requires 30 min of incubation in the patient’s arm vein, thus causing greater discomfort comparing to regular blood collection. Although no evident side effects of this device have been noted [52], the prolonged venipuncture is burdened with a minor risk of bruises, diaphoresis with hypotension, syncope and injury of muscles and nerves [54]. An alternative to in vivo CTC capture is the use of diagnostic leukapheresis [55], which enables higher CTC yields than conventional enrichment strategies using 10–20 mL of blood. 

The CTCs are a highly heterogeneous population of tumor cells. Thus, EpCAM-based enrichment technologies cannot identify all CTC sub-populations. Recently, we applied novel combinations of in vivo CTC, captured by CellCollector^®^ and downstream RT-qPCR multiplex analysis, directed to mRNA of epithelial, EMT, and stem cell markers. We found that EMT markers were frequently expressed on CTCs, which would otherwise have remained undetected by enrichment based solely on epithelial markers. This observation elucidates the need for antibody cocktails against various CTC antigens in order to capture different CTCs phenotypes [37]. On the other hand, there is a strong need to efficiently exclude cells other than CTCs from the analysis. The leukocyte common antigen—CD45 is widely used for this purpose in various CTC detection assays, including CellSearch^®^ and CellCollector. The effectiveness of non-CTCs demarcation can be strengthened by combining targeting of CD45 with other biomarkers, like those specific for myeloid cells (CD11b), tumor-associated macrophages (CD68), erythrocytes (CD235a), endothelial cells (CD146), and hematopoietic stem cells (CD34) [56,57].

Nowadays, CTC detection technologies enable the investigation of other biomarkers expressed by CTCs. Recently, attention was focused on programmed cell death ligand 1 (PD-L1) expression, given it prevents the immune system response and blocks anti-cancer immunity. In metastatic breast cancer [58], head and neck squamous cell carcinoma [59,60], and lung cancer [61] patients it has been show that combined approach of CTC detection with parallel PD-L1 immunostaining may predict the efficacy of immune checkpoint blockade therapies and the likelihood of resistance development.

When radiotherapy efficiently reduces tumor size in patients with PCa, the number of cancer cells in the circulation should also partially or totally decrease. However, non-effective RT leads to a lack of tumor response, and possibly the novel presence/increase of CTC number. Specifically, Martin et al., basing his findings on the results obtained in patients with non-small cell lung cancer (NSCLC), suggested that fractionated radiotherapy disrupts the tumor mass, thus promoting the passage of tumor cells into the circulation [62]. At an early phase of radiotherapy, when cancer cells suffer only from sub-lethal damage, the sudden increase of CTCs could, in principle, contribute to the formation of distant metastases. The paired analysis of our samples did not find any significant change in CTC counts per patient (obtained with any of the three assays) before, and after, treatment. Only a moderate tendency to decrease CTC-positive patient numbers was observed (CellSearch^®^: 15% versus 7%, EPISPOT: 49% versus 36%, CellCollector^®^: 48% versus 39%). These data do not support the hypothesis that radiotherapy in PCa leads to CTC release into the circulation. However, the time interval between the start of the RT and the second visit was approximately 256 days and detected CTCs were more likely the result of disease progression than tumor disruption following RT. For stronger evidence, immunofluorescent evaluation of phosphorylated histone H2AX (γ-H2AX) in CTCs would be helpful. This biomarker reveals the degree of double-stranded DNA breaks caused by RT. The presence of viable, γ-H2AX-positive CTCs in the peripheral circulation is the evidence of their RT-induced origin [62]. However, consecutive molecular analysis of radiotherapy-mobilized CTCs during the treatment course seems relevant because it has been suggested that such cells could return and colonize the tumor of origin. This process was described by Vilalta et al. in pre-clinical models of breast cancer as “self-seeding” [63], and could be responsible for primary tumor re-growth. It has been hypothesized that chemoattractant factors, released during radiotherapy by tumor cells, including granulocyte-macrophage colony stimulating factor (GM-CSF) [63], IL-6, or IL-8 [64], promote CTC recruitment back to the primary tumor site.

Other groups also investigated the effect of radiotherapy on CTC count. Lowes et al., using the CellSearch^®^ system in patients with PCa, observed a trend toward CTC reduction after radiotherapy, but the results were not significant, which is consistent with our results. However, the percentage of CTC-positive patients before radiotherapy was very high (73% compared with 14% in our study). Like us, they did not find any correlation between CTC numbers before, and after, therapy and PSA level, clinical tumor stage, and Gleason score [65]. Tombal et al. used nested RT-PCR for CTC detection and found a significant correlation between CTC-positivity and biochemical recurrence, and mean PSA doubling time [66]. Moreover, the response to salvage radiotherapy was observed mainly in patients without CTCs. On the other hand, a study in patients with esophageal squamous cell carcinoma found that CTC-positivity by nested RT-PCR, before therapy, is not a predictive factor for the response to radiotherapy. However, based on the observation that patients who become CTC-negative after radiotherapy had a response rate of 86%, it was concluded that CTC-positivity after treatment is a poor outcome indicator. In this study, CTC-positivity was correlated with lymph node metastases and adverse 2-year progression-free survival, but not with age, tumor size, and clinical tumor stage [67]. Similarly, Dorsey et al. found a significant decrease in CTC number (based on their elevated telomerase activity) after radiotherapy in patients with localized NSCLC. The CTC count was correlated with the clinical course and response to treatment [68]. Discrepancies between studies could be explained by the differences in patients’ status, sample size, type of assay used for the CTC count, and sampling time points.

In our study, we did not find any correlation between the CTC count (each method separately) before radiotherapy, and any of the tested clinical parameters (age, body mass index, PSA median level, Gleason score and clinical tumor stage). Theil et al. [38] tested the CellCollector^®^ in patients with PCa and detected a correlation between CTC number and tumor stage, suggesting some clinical relevance as a marker of poor prognosis. However, clinical follow-up studies are required to validate the CellCollector^®^ prognostic relevance. Moreover, CTC identification (i.e., CTC release from the nanowire) must be rapidly improved for its use in clinical trials. Surprisingly, patients with CTC-positive samples (obtained with the dual fluoro-EPISPOT assay that detect PSA-secreting cells) tended to be younger (*p* = 0.053), an observation that is inconsistent with the finding that PSA level increases with age [69]. The prognostic significance of patients’ age as an independent predictor of biochemical recurrence is still controversial. Some reports recognized advanced age as an indicator of poorer biochemical outcome [70], while others did not find any association in patients undergoing radical prostatectomy [71] or radiotherapy [72]. However, another study suggested that in high-risk younger patients, biochemical recurrence-free survival is poorer than in older patients [73]. In addition, PSA serum level was not significantly different in patients with CTC-negative and positive samples detected with the dual fluoro-EPISPOT assay. These findings suggest that the major source of serum PSA was not PSA-secreting CTCs, but rather the tumor itself. Finally, while combining three methods together, we found a significant correlation between CTC counts and patients’ BMI. The data indicate subtle [74] to strong [75,76] BMI’s effect on PCa’s incidence, aggressiveness, and mortality. The possible cause is the high concentration of leptin, insulin, insulin-like growth factor-I and low of adiponectin, which together, are likely to promote tumor growth and progression [77]. Additionally, obese men tend to have lower level of testosterone, which assures the normal prostate condition [78]. On the contrary, Schiffmann et al. described the obesity paradox in surgically-treated PCa patients, presenting a decreased risk of metastases after the treatment, possibly caused by the diabetes treatment, common for obese men [79]. The association between BMI and CTC level is not widely described, however studies on breast cancer patients show inconsistent results. Ortmann et al. found no significant relation, neither before, nor after, chemotherapy, between BMI and CTC number, suggesting they are independent prognostic factors [80], while Fayanju et al. indicated negative prognostic significance of their combination for non-obese patients exclusively [81].

## 4. Materials and Methods 

### 4.1. Study Design

This study was performed between 2013 and 2016, within the framework of the international multicenter project, “Circulating Tumor Cells as Biomarkers for Minimal Residual Disease in Prostate Cancer” (CTC-SCAN, an ERA-NET TRANSCAN project coordinated by Klaus Pantel, Hamburg). During this time, 131 patients with high-risk PCa, according to the D’Amico criteria (PSA ≥ 20 ng/mL AND/OR Gleason score on biopsy ≥ 8 AND/OR clinical tumor stage ≥ 2c), were enrolled at the Department and Clinic of Urology and Urologic Oncology at Poznan University of Medical Sciences, Poland, and at the Cancer Institute of Montpellier (ICM), France. Patients with metastases were not included in the study. The patients were examined during the first visit, before starting radiotherapy combined with hormone therapy (all 131), and at least three months after the beginning of treatment (68/131). The mean number of days (± standard deviation) between two blood samples was 256 days (±126). The experimental protocol of this study was approved by the Local Bioethics Committees at Poznan University of Medical Sciences (registered under the number 28/13), at the University Medical Centre of Montpellier and ICM (registered under the number 2013-A00523-42). All patients were informed about the study design/procedures and signed a written informed consent. Blood sampling dates, endocrine therapy, and treatment duration are presented in Table S1.

### 4.2. CTC Isolation and Detection

For CTC detection, using the CellSearch^®^ system (Silicon Biosystem, Menarini, Florence, Italy) and the dual fluoro-EPISPOT^PSA/FGF2^ assay, 10mL of blood was collected in CellSave^®^ tubes, and EDTA tubes, respectively. For CTC detection with CellCollector^®^ CANCER01 (Gilupi GmbH, Potsdam, Germany), the device was placed directly in the arm vein for 30 min. 

Within 24 h after blood draw, the samples, collected in CellSave^®^ tubes in Poznan, were shipped at room temperature to the Laboratory of Rare Human Circulating Cells (LCCRH) at the University Medical Center of Montpellier, France, where the samples were analyzed on arrival day.

#### 4.2.1. CellSearch^®^ System 

The FDA-cleared CellSearch^®^ system has been used as a “gold standard” for CTC detection in patients with metastatic PCa for almost one decade [82,83]; however, in this project, CellSearch^®^ was used to detect CTCs in patients without metastases. The CellSearch^®^ Circulating Epithelial Cell Kit (Silicon Biosystems, Menarini) uses 7.5 mL of blood for CTC enrichment and enumeration. The CellSearch^®^ system is based on magnetic beads (ferrofluid) coated with antibodies against EpCAM for CTC enrichment. EpCAM-positive cells are captured and then immunostained with antibodies against cytokeratins (CK 8, 18 and 19) and with DAPI (nuclear staining). EpCAM- and CK-positive and CD45 (common leukocyte antigen)-negative cells are identified as CTCs [41,82].

#### 4.2.2. Dual Fluoro-EPISPOT^PSA/FGF2^ Assay

CTCs from 10 mL of blood samples were first enriched by negative selection using 20 µL of RosetteSep^TM^ Human Circulating Epithelial Tumor Cell Enrichment Cocktail (STEMCELL Technologies, Vancouver, Canada) per 1mL of blood. After incubation and phase separation with 1.073 density gradient, CTCs were collected from the interphase and washed twice. 

For this study, the dual fluoro-EPISPOT**^PSA/FGF2^** assay was used to detect CTCs that secrete PSA and fibroblast growth factor 2 (FGF2, an important self-renewal regulator of normal and cancer stem cells), as previously described [39,40]. We previously showed that this assay can detect CTCs that secrete PSA and FGF2 from blood samples of patients with PCa [36]. Briefly, after negative enrichment, CTCs were cultured at 37 °C and 5% CO_2_ on nitrocellulose membranes pre-coated with 1.04 µg/µL anti-PSA H50 antibody (provided by Prof Hans Lilja, Department of Biotechnology, University of Turku, Finland) and 0.5 µg/µL anti-FGF2 500-M38 antibody (Peprotech), diluted in PBS and blocked with 5% BSA/PBS. After 48 h, PSA and FGF2, produced by viable cells and captured by the primary antibodies, were detected by incubation with fluorochrome-conjugated anti-PSA-H117-A555 antibody (1.0 µg/µL; provided by Prof. Hans Lilja, Department of Biotechnology, University of Turku, Finland) and anti-FGF2 500-P18Bt antibody labeled with biotin (0.5 µg/µL; Peprotech) and then with 1:20 anti-biotin-FITC antibody (Miltenyi Biotec) diluted in 0.5% bovine serum albumin (BSA)/PBS. After washing, PSA and FGF2 immuno-spots were imaged and counted using a fluorescent microscope, and a video-camera imaging and computer-assisted analysis system (KS ELISPOT, Carl Zeiss Vision) and also the C.T.L. ELISPOT reader [84,85].

The LNCap (PCa) and NBTII cell lines from the American Type Culture Collection (ATCC) were used as positive control for PSA, and FGF2 expression/secretion, respectively. LNCaP cells (ATCC^®^ CRL-1740™) were cultured in RPMI 1640 medium (Gibco), 10% fetal bovine serum (FBS) (Sigma Aldrich) and antibiotics (Sigma Aldrich). NBTII cells (ATCC^®^ CRL-1655^TM^) were grown in DMEM medium with GlutaMAX (Gibco), 10% FBS (Sigma Aldrich), and antibiotics (Sigma Aldrich). At 80% confluence in 75 cm^2^ flasks, the cells were trypsinized (0.25% trypsin/EDTA, Gibco), washed, and counted in order to seed 2000 cells/well (two wells) in each plate. For the dual fluoro-EPISPOT assay, LNCap, NBTII and enriched CTCs were cultured in RPMI 1640 medium (Sigma Aldrich) with 10% FBS (Sigma Aldrich), antibiotics (Sigma Aldrich), 1% L-glutamine (Sigma Aldrich), and 1% Insulin-Transferrin-Selenium (Gibco).

#### 4.2.3. CellCollector^®^

The CellCollector^®^ CANCER01 (Gilupi GmbH) allows the in vivo capture and detection of CTCs [52]. The device is composed of medical stainless-steel wire, coated with polycarboxylate hydrogel and functionalized with anti-EpCAM antibodies. The CellCollector^®^ was placed in the vein of the arm using a standard cannula (32 mm) for 30 min. After its removal, the cells captured on the wire were fixed in cold acetone for 10 min, permeabilized in 0.1% Triton X-100/PBS for 10 min, and blocked in 3% BSA/PBS for 30 min. Then, the cells were incubated for 1h with fluorochrome-conjugated antibodies against PanCK (A488 antibody cocktail, 1:50; eBioscience, Exbio) and PSA (H117-A555 antibody, 1:80; provided by Prof Hans Lilja, Department of Biotechnology, University of Turku, Finland), all diluted in 3% BSA/PBS. To confirm the absence of leukocytes, the cells were incubated with the anti-CD45-A647 antibody (1:25; Exbio), diluted in 3% BSA/PBS. The nuclei were stained with Hoechst 33258 (Sigma Aldrich) for 10 min. The images were then obtained using a fluorescent microscope (Axio Imager 2, Carl Zeiss) and a 20× objective and were analyzed with AxioVision 4.8 (Carl Zeiss). The cells were identified as CTCs using the following criteria: Intact morphology, polymorphic, diameter ≥4 µm, CK- and/or PSA-positivity, Hoechst 33258-positivity, and CD-45 negativity.

### 4.3. Statistical Analysis

For analysis with clinical-pathological variables, CTC counts were stratified as negative (no CTC detected) or positive (at least one CTC detected). To test for correlations between CTC positivity and clinical-pathological variables, the Wilcoxon-test was used for continuous variables, and the chi-square (likelihood) test for categorical variables. To compare the CTC counts before, and at least three months after, the initiation of radiotherapy, the McNemar’s test was used. To assess the clinical risk of biochemical progression after radiotherapy, patients with negative and positive CTC counts were compared using the chi-square (likelihood) test. All tests were two-sided and a *p*-value < 0.05 was considered to be significant. Data were analyzed using RStudio (version 1.1.383), an integrated development environment for R (version 3.4.2). The readxl package (version 1.0.0) was used to load the Excel file containing the experimental data into R, and the dplyr package (version 0.7.4) for data manipulation, the MASS package for the chi-square likelihood test, and the Stats package (version 3.4.2) for the Wilcoxon test. 

## 5. Conclusions

Combining different CTC assays improved CTC detection rates in patients with non-metastatic, high-risk PCa before, and after, treatment. Our findings do not support the hypothesis that radiotherapy leads to cancer cell release in the circulation. Additional clinical trials are required before the implementation of CTC detection in the standard clinical practice of patients with PCa receiving radiotherapy. Moreover, a better molecular characterization of CTCs might provide additional information on the survival mechanisms implemented during their rough passage in the bloodstream and on the biology behind extravasation in distant organs [37,86].

## Figures and Tables

**Table 1:** Clinical characteristics of the patients.

Parameter	Overall	CellSearch^®^ System			EPISPOT			CellCollector^®^			Combined		
		CTC Negative	CTC Positive	*p*-Value	CTC Negative	CTC Positive	*p*-Value	CTC Negative	CTC Positive	*p*-Value	CTC Negative	CTC Positive	*p*-Value
Patients, *n* (%)	131	94 (82)	20 (18)		67 (54)	57 (46)		60 (48)	66 (52)		16 (12)	115 (88)	
Age, Median (IQR), Min–Max	68.5, (64.25–72), 51–89	68 (64–72) 51–80	70.5 (66.75–72.75) 60–89	0.15	69 (66–72) 53–89	66 (63–72) 51–79	0.053	69 (64–72) 51–89	67 (64.25–72) 53–78	0.50	69, (67.5–72.25), 59–80	68, (64–72), 51–89	0.22
BMI, Median (IQR), Min–Max	27.61, (25.6–29.8), 20.45–46.17	27.4, (25.5–29.7), 20.8–46.17	28.01, (26.8–29.4), 23.2–41.9	0.37	27.13, (25.5–29.5), 20.8–41.91	27.7, (26.03–30.67), 20.45–37.18	0.33	27.46, (25.24–29.11), 20.45–37.18	27.68, (26.17–31.7), 21.51–46.17	0.19	25.59, (23.5–26.9), 20.8–29.4	27.68, (26.12–30.66), 20.45–46.17	0.0036
PSA, Median (IQR), Min–Max	27.85, (15–35–40.75), 0.5–191	28.2 (20–42) 3.89–191	25.6 (7–09–40.25) 0.5–172	0.26	26.795 (10.365–41.15) 0.5–172	28.2 (17.6–40) 5–191	0.23	25 (10.5–41.1) 2.5–191	28.5 (22.5–37) 0.5–136.9	0.23	24.83, (11.78–38.95), 3.9–66	28.05, (15.625–40.75), 0.5–191	0.49
Biopsy Gleason score; *n* (%)													
3+3	30 (23)	22 (23.9)	4 (20)	0.77	13 (19.7)	15 (26.8)	0.069	13 (22)	17 (25.8)	0.14	3 (18.8)	27 (23.9)	0.35
3+4	40 (31)	30 (32.6)	4 (20)		18 (27.3)	20 (35.7)		19 (32.2)	21 (31.8)		4 (25)	36 (31.8)	
4+3	24 (19)	19 (20.7)	2 (10)		10 (15.2)	12 (21.4)		10 (16.9)	12 (18.2)		4 (25)	20 (17.7)	
≥4+4	35 (28)	21 (22.8)	10 (50)		25 (37.8)	9 (16.1)		17 (28.9)	16 (24.2)		5 (31.2)	30 (26.6)	
Clinical T stage; *n* (%)													
T1a	1 (0.8)	0 (0)	0 (0)	0.64	0 (0)	1 (1.8)	0.078	0 (0)	1 (1.6)	0.24	0 (0)	1 (0.9)	0.95
T1c	76 (60.3)	56 (62.2)	9 (47.4)		36 (55.4)	35 (63.6)		31 (53.4)	45 (70.3)		10 (62.5)	66 (60)	
T2a	10 (7.9)	6 (6.7)	3 (15.8)		4 (6.2)	6 (10.9)		7 (12.1)	3 (4.7)		1 (6.2)	9 (8.2)	
T2b	3 (2.4)	3 (3.3)	0 (0)		2 (3.1)	0 (0)		1 (1.7)	2 (3.1)		0 (0)	3 (2.7)	
T2c	23 (18.3)	16 (17.8)	4 (21.1)		13 (20)	10 (18.2)		12 (20.7)	9 (14.1)		3 (18.8)	20 (18.2)	
T3a	12 (9.5)	8 (8.9)	3 (15.8)		10 (15.4)	2 (3.6)		6 (10.3)	4 (6.2)		2 (12.5)	10 (9.1)	
T3b	1 (0.8)	1 (1.1)	0 (0)		0 (0)	1 (1.8)		1 (1.7)	0 (0)		0 (0)	1 (0.9)	

BMI, body mass index; PSA, prostate specific antigen.

**Table 2:** Number of blood samples tested with each circulating tumor cells (CTC) enumeration method.

Samples Tested	CellSearch^®^	EPISPOT	CellCollector^®^
No. of samples tested at baseline	114	124	126
No. of samples tested after radiotherapy	63	68	64
No. of samples tested at both time points	53	62	64

## Supplementary Materials

The following are available online at https://www.mdpi.com/2072-6694/11/6/802/s1, Table S1: Blood sampling dates, endocrine therapy, and treatment duration.
