# Supplementary material for: Analysis of Circulating Tumor Cells in Patients with Non-Metastatic High-Risk Prostate Cancer before and after Radiotherapy Using Three Different Enumeration Assays
Source: Cancers (Basel). 2019 Jun 10;11(6):802. doi: 10.3390/cancers11060802 (PMC6627099; doi:10.3390/cancers11060802)
Supplement: Supplementary file 1 [file cancers-11-00802-s001.pdf]

Supplementary Materials: Analysis of Circulating Tumor Cells in Patients with Non-Metastatic High-Risk Prostate Cancer before and after Radiotherapy Using Three Different Enumeration Assays

Joanna Budna-Tukan , Monika Świerczewska, Martine Mazel, Wojciech A. Cieślikowski, Agnieszka Ida, Agnieszka Jankowiak, Andrzej Antczak, Michał Nowicki, Klaus Pantel, David Azria, Maciej Zabel and Catherine Alix-Panabières

Table S1. Blood sampling dates, endocrine therapy and treatment duration.

Patients	Date of Inclusion	Radiotherapy (RT)		Samples			Endocrine Therapy (ET)			
		Start RT	End RT	Sample n°1	Sample n°2	Days between the Two Blood Draws	Name	Date of Initiation	Duration (Months)	Situation
1	2014-08-05	2014-09-17	2014-11-12	2014-08-05	2014-12-09	126	Enantone 11.25 mg	2014-08-07	18	Concomitant
2	2014-08-05	Not done	Not done	2014-08-05	N/A	N/A	LH-RH agonist	Unknown	36	N/A
3	2014-08-19	2014-09-17	2014-11-06	2014-08-19	2014-12-16	119	Enantone 30 mg	2014-08-19	24	Concomitant
4	2014-11-19	2014-12-04	2015-01-28	2014-11-19	2015-03-11	112	Decapeptyl 22.5 mg	2014-11-19	24	Concomitant
5	2015-01-06	2015-01-19	2015-03-10	2015-01-07	2015-04-23	106	Enantone 30 mg	2015-01-07	24	Concomitant
6	2015-01-06	2015-01-15	2015-03-05	2015-01-07	2015-04-22	105	Casodex 50 mg<br>Enantone 30 mg	2015-01-07	12	Concomitant
7	2015-01-13	2015-02-02	2015-03-25	2015-01-14	2015-06-03	140	Decapeptyl 22.5 mg	2015-01-14	36	Concomitant
8	2015-03-19	2015-04-07	2015-06-04	2015-03-25	2015-07-08	105	Casodex 50 mg<br>Decapeptyl 22.5 mg	2015-03-25	18	Concomitant
9	2015-03-25	2015-04-08	2015-06-08	2015-03-25	2015-06-08	75	Enantone 30 mg	2015-03-25	12	Concomitant
10	2015-04-08	2015-04-22	2015-06-19	2015-04-08	2015-07-22	105	Enantone 30 mg	2015-04-08	36	Concomitant
11	2015-05-26	2015-06-09	2015-07-31	2015-05-27	2015-09-16	112	Enantone 30 mg	2015-05-27	18	Concomitant
12	2015-06-24	2015-07-15	2015-09-04	2015-06-24	2015-10-14	112	Casodex 50 mg<br>Decapeptyl 22.5 mg	2015-06-24	24	Concomitant
13	2015-09-08	2015-12-03	2016-01-28	2015-09-25	2016-03-03	160	Enantone 11.25 mg then Zoladex on 10/2016	2015-09-22	15 months then refused to continue	Concomitant
14	2015-09-17	2015-10-14	2015-12-04	2015-09-23	2016-01-20	119	Decapeptyl 22.5 mg	2015-09-23	24	Concomitant
15	2015-10-16	2015-11-09	2015-12-31	2015-10-28	2016-02-03	98	Enantone 11.25 mg	2015-10-28	18	Concomitant
16	2015-10-16	2015-11-12	2016-01-05	2015-10-21	2016-02-11	113	Enantone 30 mg	2015-10-21	24	Concomitant
17	2015-12-01	2016-01-18	2016-03-09	2015-12-02	2016-04-18	138	Enantone 30 mg	2015-12-02	12	Concomitant
18	2016-01-13	2016-02-18	2016-04-15	2016-02-18	2016-05-18	90	Enantone 30 mg	2016-01-13	36	Concomitant
19	2016-02-23	2016-03-10	2016-05-03	2016-03-10	N/A	N/A	Enantone 11.25 mg	2016-02-24	12	Concomitant
20	2016-03-01	Unknown	Unknown	2016-03-01	N/A	N/A	Casodex 50 mg<br>Enantone 11,25 mg	2016-03-02	Unknown	Unknown

21	2016-03-07	2016-03-16	2016-05-17	2016-03-02	N/A	N/A	Casodex 50 mg<br>Enantone 30 mg	2016-03-02	12	Concomitant
22	2016-03-23	2016-04-08	2016-06-03	2016-03-23	N/A	N/A	Enantone 30 mg	2016-03-23	36	Concomitant
23	2016-04-07	2016-04-28	2016-06-28	2016-04-06	2016-08-02	118	Enantone 11.25 mg	2016-04-06	12	Concomitant
24	2016-06-29	2016-07-26	2016-09-20	2016-06-29	2016-11-02	126	Enantone 11.25 mg	2016-06-29	6	Concomitant
25	2016-10-11	2017-01-09	2017-03-01	2016-10-11	2017-03-29	169	Enantone 30 mg<br>Casodex/placebo<br>ARN-509/placebo	2017-11-08<br>2017-11-08<br>2016-10-17	36<br>4<br>36	Concomitant
26	2016-10-18	Unknown	Unknown	2016-10-18	N/A	N/A	Unknown	Unknown	Unknown	Unknown
27	2013-12-12	2014-08-07	2014-09-30	2013-12-12	N/A	N/A	Zoladex 10.8 mg	2013-12-18	36	Concomitant
28	2013-12-12	2013-11-05	2014-01-03	2013-12-12	N/A	N/A	Eligard 45 mg	2013-12-18	36	Concomitant
29	2013-12-12	2014-04-01	2014-05-27	2013-12-12	2014-06-30	200	Diphereline 11.25 mg	2013-12-18	36	Concomitant
30	2014-02-18	2014-07-23	2014-09-15	2014-02-18	2014-11-13	268	Zoladex 10.8	2014-02-23	36	Concomitant
31	2014-02-18	2014-06-06	2014-08-01	2014-02-18	2014-09-10	204	Diphereline 11.25 mg	2014-02-23	36	Concomitant
32	2014-02-18	2014-08-14	2014-09-29	2014-02-18	N/A	N/A	Eligard	2014-02-23	36	Concomitant
33	2014-04-01	2014-08-11	2014-10-01	2014-04-01	2014-11-13	226	Eligard 22.5 mg	2014-04-07	36	Concomitant
34	2014-04-22	2014-07-18	2014-09-15	2014-04-22	2015-04-08	351	Eligard 22.5 mg	2014-04-28	36	Concomitant
35	2014-04-22	2014-07-18	2014-09-15	2014-04-22	2014-10-29	190	Zoladex 10.8	2014-04-28	36	Concomitant
36	2014-04-28	2014-08-04	2014-09-22	2014-04-28	2014-12-02	218	Eligard 22.5 mg	2014-05-07	36	Concomitant
37	2014-05-14	2014-08-29	2014-10-23	2014-05-14	2015-03-12	302	Diphereline 11.25 mg	2014-05-20	36	Concomitant
38	2014-05-28	2014-09-10	2014-10-23	2014-05-28	2014-12-09	195	Eligard 22.5 mg	2014-06-07	36	Concomitant
39	2014-05-28	2014-09-04	2014-10-15	2014-05-28	2014-12-09	195	Eligard 22.5 mg	2014-06-07	36	Concomitant
40	2014-05-28	2014-08-26	2014-10-16	2014-05-28	2014-12-16	202	Diphereline 11.25 mg	2014-06-07	36	Concomitant
41	2014-06-11	2014-09-17	2014-10-28	2014-06-11	2015-03-12	274	Eligard 45 mg	2014-06-18	36	Concomitant
42	2014-06-11	2014-09-16	2014-11-05	2014-06-11	N/A	N/A	Eligard 22.5 mg	2014-06-18	36	Concomitant
43	2014-06-26	2014-10-06	2014-11-16	2014-06-26	2015-03-12	259	Zoladex 10.8	2014-07-02	36	Concomitant
44	2014-06-26	2014-10-03	2014-11-26	2014-06-26	2015-07-21	390	Eligard 22.5 mg	2014-07-02	36	Concomitant
45	2014-07-15	2014-10-17	2014-12-17	2014-07-15	N/A	N/A	Diphereline 11.25 mg	2014-07-20	36	Concomitant
46	2014-08-13	2014-12-04	2015-01-27	2014-08-13	2015-07-21	342	Eligard 22.5 mg	2014-08-20	36	Concomitant
47	2014-08-13	died before RT		2014-08-13	N/A	N/A	Diphereline 11.25 mg	2014-08-20	36	Concomitant
48	2014-09-02	2015-12-30	2015-03-18	2014-09-02	2015-07-21	322	Diphereline 11.25 mg	2014-09-09	36	Concomitant
49	2014-09-02	2015-01-12	2015-03-05	2014-09-02	2015-07-21	322	Eligard 45 mg	2014-09-09	36	Concomitant
50	2014-09-10	2014-12-29	2015-02-28	2014-09-10	2015-07-13	306	Zoladex 10.8 mg	2014-09-17	36	Concomitant
51	2014-09-10	2014-12-19	2015-02-13	2014-09-10	2015-08-27	351	Zoladex 10.8 mg	2014-09-17	36	Concomitant
52	2014-09-30	2015-01-16	2015-03-30	2014-09-30	2015-08-27	331	Diphereline 11.25 mg	2014-10-06	36	Concomitant
53	2014-10-20	2015-01-28	2015-03-19	2014-10-20	2015-09-16	331	Zoladex 10.8 mg	2014-10-27	36	Concomitant
54	2014-10-20	2015-02-16	2015-04-17	2014-10-20	N/A	N/A	Zoladex 10.8 mg	2014-10-27	36	Concomitant
55	2014-10-20	no RT		2014-10-20	N/A	N/A	Firmagon	2014-10-27	36	Concomitant
56	2014-10-29	no RT		2014-10-29	N/A	N/A	no ET	N/A	36	Concomitant
57	2014-11-13	2015-02-17	2015-05-15	2014-11-13	2015-07-22	251	Diphereline 11.25 mg	2014-11-20	36	Concomitant

58	2014-12-02	2015-02-16	2015-04-09	2014-12-02	2015-07-08	218	Zoladex 10.8 mg	2014-12-09	36	Concomitant
59	2014-12-02	2015-03-16	2015-04-22	2014-12-02	2015-09-03	275	Diphereline 11.25 mg	2014-12-09	36	Concomitant
60	2014-12-16	2015-03-31	2015-05-21	2014-12-16	2015-09-06	264	Zoladex 10.8 mg	2014-12-22	36	Concomitant
61	2015-02-17	2015-05-29	2015-07-27	2015-02-17	2015-10-28	253	Lucrin 3.75 mg	2015-02-24	36	Concomitant
62	2017-02-17	2015-08-25	2015-10-14	2015-02-17	2015-11-03	259	Eligard 22.5 mg	2017-02-24	36	Concomitant
63	2015-03-10	2015-05-06	2015-06-17	2015-03-10	N/A	N/A	Zoladex 10.8 mg	2015-03-17	36	Concomitant
64	2015-03-10	2015-07-24	2015-09-16	2015-03-10	2015-11-24	259	Diphereline 11.25 mg	2015-03-17	36	Concomitant
65	2015-03-30	2015-09-18	2015-10-05	2015-03-30	N/A	N/A	Eligard 22.5 mg	2015-04-07	36	Concomitant
66	2015-03-30	2015-05-28	2015-07-08	2015-03-30	2015-10-20	204	Diphereline 11.25 mg	2015-04-07	36	Concomitant
67	2015-03-30	2015-07-07	2015-09-03	2015-03-30	2015-11-24	239	Diphereline 11.25 mg	2015-04-07	36	Concomitant
68	2015-04-07	2015-05-25	2015-07-06	2015-04-07	2015-12-01	238	Diphereline 11.25 mg	2015-04-14	36	Concomitant
69	2015-04-29	2015-08-27	2015-10-24	2015-04-29	2016-05-18	385	Diphereline 11.25 mg	2015-05-05	36	Concomitant
70	2015-05-05	2015-08-25	2015-10-28	2015-05-05	2016-02-02	273	Diphereline 11.25 mg	2015-05-12	36	Concomitant
71	2015-05-13	2015-06-29	2015-07-31	2015-05-13	2016-02-02	265	Zoladex 10.8 mg	2015-05-20	36	Concomitant
72	2015-05-26	no RT		2015-05-26	N/A	N/A	no ET	N/A	36	Concomitant
73	2015-05-26	2015-09-04	2015-11-09	2015-05-26	2016-05-18	358	Eligard 22.5 mg	2015-06-03	36	Concomitant
74	2015-07-06	2016-01-11	2016-02-17	2015-07-06	2017-02-21	596	Zoladex 10.8 mg	2015-07-13	36	Concomitant
75	2015-07-06	2015-09-11	2015-11-14	2015-07-06	2017-02-21	596	Eligard 22.5 mg	2015-07-13	36	Concomitant
76	2015-08-19	no RT		2015-08-19	2017-02-21	552	Zoladex 10.8 mg	2015-08-26	36	Concomitant
77	2015-09-10	2016-01-07	2016-03-04	2015-09-10	N/A	N/A	Zoladex 10.8 mg	2015-09-17	36	Concomitant
78	2015-09-10	no RT		2015-09-10	N/A	N/A	no ET	N/A	36	Concomitant
79	2015-09-17	2015-12-22	2016-02-24	2015-09-17	2017-02-21	523	Diphereline 11.25 mg	2015-09-24	36	Concomitant
80	2015-10-08	2016-01-29	2016-04-06	2015-10-08	N/A	N/A	Eligard 22.5 mg	2015-10-15	36	Concomitant
81	2015-10-08	no RT		2015-10-08	N/A	N/A	Eligard 22.5 mg	2015-10-15	36	Concomitant
82	2015-10-13	2016-02-16	2016-04-27	2015-10-13	2016-05-18	218	Eligard 22.5 mg	2015-10-20	36	Concomitant
83	2015-10-20	no RT		2015-10-20	N/A	N/A	Eligard 22.5 mg	2015-10-27	36	Concomitant
84	2015-10-28	no RT		2015-10-28	N/A	N/A	no ET	N/A	36	Concomitant
85	2015-10-28	no RT		2015-10-28	2017-03-21	510	Zoladex 10.8 mg	2015-11-04	36	Concomitant
86	2015-10-28	2016-02-09	2016-04-12	2015-10-28	2017-03-21	510	Zoladex 10.8 mg	2015-11-04	36	Concomitant
87	2015-11-03	no RT		2015-11-03	N/A	N/A	Eligard 22.5 mg	2015-11-10	36	Concomitant
88	2015-11-24	2016-04-05	2016-06-15	2015-11-24	N/A	N/A	Zoladex 10.8 mg	2015-12-01	36	Concomitant
89	2015-12-01	no RT, nodes metastasis		2015-12-01	N/A	N/A	Eligard 45 mg	2015-12-08	36	Concomitant
90	2015-12-01	2016-03-08	2016-04-11	2015-12-01	N/A	N/A	Eligard 45 mg	2015-12-08	36	Concomitant
91	2016-01-14	no RT		2016-01-14	N/A	N/A	Diphereline 11.25 mg	2016-01-21	36	Concomitant
92	2016-01-14	Cyberknife		2016-01-14	N/A	N/A	Eligard 22.5 mg	2016-01-21	36	Concomitant
93	2016-01-14	2016-03-09	2016-04-20	2016-01-14	2017-03-21	432	Eligard 45 mg	2016-01-22	36	Concomitant
94	2016-01-25	no RT		2016-01-25	N/A	N/A	Diphereline 11.25 mg	2016-02-03	36	Concomitant
95	2016-01-25	no RT, nodes metastasis		2016-01-25	N/A	N/A	Diphereline 11.25 mg	2016-02-03	36	Concomitant
96	2016-01-25	2016-02-26	2016-05-18	2016-01-25	N/A	N/A	Diphereline 11.25 mg	2016-02-03	36	Concomitant

97	2016-02-08	2016-03-27	2016-05-20	2016-02-08	N/A	N/A	Eligard 22.5 mg	2016-02-16	36	Concomitant
98	2016-02-15	2016-04-25	2016-06-15	2016-02-15	N/A	N/A	Zoladex 10.8 mg	2016-02-22	36	Concomitant
99	2016-02-15	2014-01-29	2014-03-28	2016-02-15	N/A	N/A	Diphereline 11.25 mg	2016-02-22	36	Concomitant
100	2016-02-23	2016-04-07	2016-05-31	2016-02-23	N/A	N/A	Zoladex 10.8 mg	2016-03-02	36	Concomitant
101	2016-03-16	2016-05-16	2016-06-29	2016-03-16	2017-03-21	370	Eligard 22.5 mg	2016-03-23	36	Concomitant
102	2016-03-23	2016-05-16	2016-09-15	2016-03-23	N/A	N/A	Eligard 22.5 mg	2016-03-30	36	Concomitant
103	2016-03-29	2016-09-08	2016-11-04	2016-03-29	N/A	N/A	Diphereline 11.25 mg	2016-04-05	36	Concomitant
104	2016-03-31	2016-07-07	2016-09-08	2016-03-31	N/A	N/A	Diphereline 11.25 mg	2016-04-07	36	Concomitant
105	2016-03-31	2016-09-08	2016-11-02	2016-03-31	N/A	N/A	Zoladex 10.8 mg	2016-04-07	36	Concomitant
106	2016-04-06	2016-09-07	2016-11-05	2016-04-06	2017-03-21	349	Diphereline 11.25 mg	2016-04-13	36	Concomitant
107	2016-04-13	2016-07-08	2016-09-22	2016-04-13	N/A	N/A	Eligard 22.5 mg	2016-04-20	36	Concomitant
108	2016-04-13	2016-08-05	2016-09-28	2016-04-13	N/A	N/A	Diphereline 11.25 mg	2016-04-20	36	Concomitant
109	2016-04-20	2016-08-05	2016-09-27	2016-04-20	2017-03-28	342	Eligard 22.5 mg	2016-04-27	36	Concomitant
110	2016-04-20	2016-08-01	2016-09-30	2016-04-20	2017-03-21	335	Firmagon 80 mg	2016-04-27	36	Concomitant
111	2016-04-28	2016-07-29	2016-09-21	2016-04-28	2017-02-28	306	Eligard 22.5 mg	2016-05-06	36	Concomitant
112	2016-04-28	no RT		2016-04-28	2017-03-07	313	no ET	N/A	36	Concomitant
113	2016-04-28	no RT		2016-04-28	2017-03-07	313	Diphereline 11.25	2016-05-06	36	Concomitant
114	2016-05-26	metastasis		2016-05-26	N/A	N/A	Eligard 22.5 mg	2016-06-04	36	Concomitant
115	2016-06-07	metastasis		2016-06-07	N/A	N/A	Eligard 22.5 mg	2016-06-13	36	Concomitant
116	2016-07-05	metastasis		2016-07-05	N/A	N/A	Diphereline 11.25 mg	2016-07-12	36	Concomitant
117	2016-07-18	2016-04-20	2016-05-24	2016-07-18	N/A	N/A	Diphereline 11.25 mg	2016-07-25	36	Concomitant
118	2016-07-18	2016-09-23	2016-11-23	2016-07-18	N/A	N/A	Eligard 22.5 mg	2016-07-25	36	Concomitant
119	2016-07-18	metastasis		2016-07-18	N/A	N/A	Eligard 22.5 mg	2016-07-25	36	Concomitant
120	2016-07-18	metastasis		2016-07-18	N/A	N/A	Diphereline 11.25 mg	2016-07-25	36	Concomitant
121	2016-08-02	metastasis		2016-08-02	N/A	N/A	no ET	N/A	36	Concomitant
122	2016-08-16	2016-10-13	2016-12-15	2016-08-16	2017-03-28	224	Zoladex 10.8 mg	2016-08-23	36	Concomitant
123	2016-09-20	2016-12-15	2017-01-17	2016-09-20	2017-03-28	189	Reseligo 10.8 mg	2016-09-27	36	Concomitant
124	2016-09-20	2017-06-12	2017-08-04	2016-09-20	N/A	N/A	Diphereline 11.25 mg	2016-09-27	36	Concomitant
125	2016-10-04	metastasis		2016-10-04	N/A	N/A	Firmagon 80 mg	2016-10-11	36	Concomitant
126	2016-10-04	2017-03-21	2017-05-24	2016-10-04	N/A	N/A	Diphereline 11.25 mg	2016-10-11	36	Concomitant
127	2016-10-18	no RT		2016-10-18	N/A	N/A	Diphereline 11.25 mg	2016-10-25	36	Concomitant
128	2016-11-08	2017-01-30	2017-04-03	2016-11-08	N/A	N/A	Diphereline 11.25 mg	2016-11-15	36	Concomitant
129	2016-11-08	2017-03-28	2017-05-19	2016-11-08	N/A	N/A	Diphereline 11.25 mg	2016-11-15	36	Concomitant
130	2016-11-29	2017-03-14	2017-04-20	2016-11-29	N/A	N/A	Eligard 22.5 mg	2016-12-05	36	Concomitant
131	2016-11-29	2017-03-06	2017-04-28	2016-11-29	N/A	N/A	Eligard 45 mg	2016-12-05	36	Concomitant

© 2019 by the authors. Licensee MDPI, Basel, Switzerland. This article is an open access article distributed under the terms and conditions of the Creative Commons Attribution (CC BY) license (http://creativecommons.org/licenses/by/4.0/).
